# Microglia-derived metabolites as novel signaling molecules: regulating neural circuit function, behavior, and their role in psychiatric disorders

**DOI:** 10.3389/fncel.2026.1718657

**Published:** 2026-03-11

**Authors:** Jiatai Li, Yonghou Zhao

**Affiliations:** Heilongjiang University of Chinese Medicine, Harbin, Heilongjiang, China

**Keywords:** interstitial space, itaconate, kynurenine, lactate, metabolites, microglia, neuro-immune-metabolic axis, succinate

## Abstract

Microglia, central immune and metabolic regulators, release metabolites that act as signaling molecules into the interstitial space within the neuro-immune-metabolic axis (NIMA). This review synthesizes evidence on how microglia-derived metabolites modulate neural circuit function—affecting synaptic plasticity, network oscillations, and behavior—and how their dysregulation contributes to psychiatric disorders. We highlight the dynamic role of the interstitial space in shaping metabolite signaling and discuss therapeutic strategies targeting this axis. Reconceptualizing microglial metabolites as active circuit modulators offers novel insights into psychiatric pathophysiology and potential metabolic interventions.

## 1 Introduction

### 1.1 The global burden of psychiatric disorders and current therapeutic limitations

Psychiatric disorders represent a leading cause of disability and loss of life worldwide, imposing a tremendous public health burden (Yang et al., 2025). Major depressive disorder (MDD), affecting an estimated 280 million people globally with a lifetime prevalence of 10%–20% and a female-to-male ratio of approximately 2:1, stands as the foremost contributor to functional disability (Nayak et al., 2014; Yang et al., 2025). Schizophrenia (SZ), while less prevalent (∼0.3%–0.7%), typically manifests in adolescence or early adulthood and is associated with a 10–20-year reduction in life expectancy, conferring profound long-term impacts on patients, families, and society (Yang et al., 2025). Despite the partial efficacy of current therapies (e.g., antipsychotics, antidepressants), a substantial proportion of patients exhibit inadequate response or persistent core symptoms—particularly cognitive and negative symptoms—highlighting fundamental gaps in our understanding of their underlying pathophysiology (Chausse et al., 2021; Jauhar et al., 2022).

### 1.2 The emergence of a new paradigm and the core thesis of this review

This therapeutic impasse necessitates novel mechanistic frameworks. The integrated perspective of the “neuro-immune-metabolic axis (NIMA)” (Bohlen et al., 2017) has recently emerged as a transformative lens through which to re-examine psychiatric diseases (Bernier et al., 2020a,b). Within this framework, the role of microglia, the brain’s resident immune cells which constitute approximately 5%–10% of all cells in the central nervous system (Martins-Ferreira et al., 2025), is being fundamentally redefined: they are not merely immune sentinels but also active metabolic regulators and sources of signaling molecules (Bernier et al., 2020b; Erny et al., 2021; Liu and Zhou, 2024). This review posits a central thesis: under stress or pathological conditions, activated microglia undergo metabolic reprogramming, releasing a specific repertoire of metabolites—such as lactate, succinate, itaconate, and kynurenine. Critically, these molecules function not merely as metabolic waste or energy substrates but as novel chemical messengers. They are released into the brain’s dynamic interstitial space—a compartment far from being a passive conduit. Through dynamic fluctuations in its volume, composition, and diffusion properties, this space actively modulates the concentration, spatiotemporal range, and persistence of these signaling metabolites (Dogan et al., 2018; Holland et al., 2018; Hrabetova et al., 2018). Consequently, these microglia-derived metabolic signals directly regulate neuronal excitability, synaptic plasticity, and network oscillations, ultimately shaping behavioral outputs. Therefore, dysregulation of the “microglial metabolite–interstitial space–neural circuit” communication axis constitutes a pivotal mechanism driving neural circuit dysfunction in psychiatric disorders.

### 1.3 Review roadmap

**FIGURE 1:**
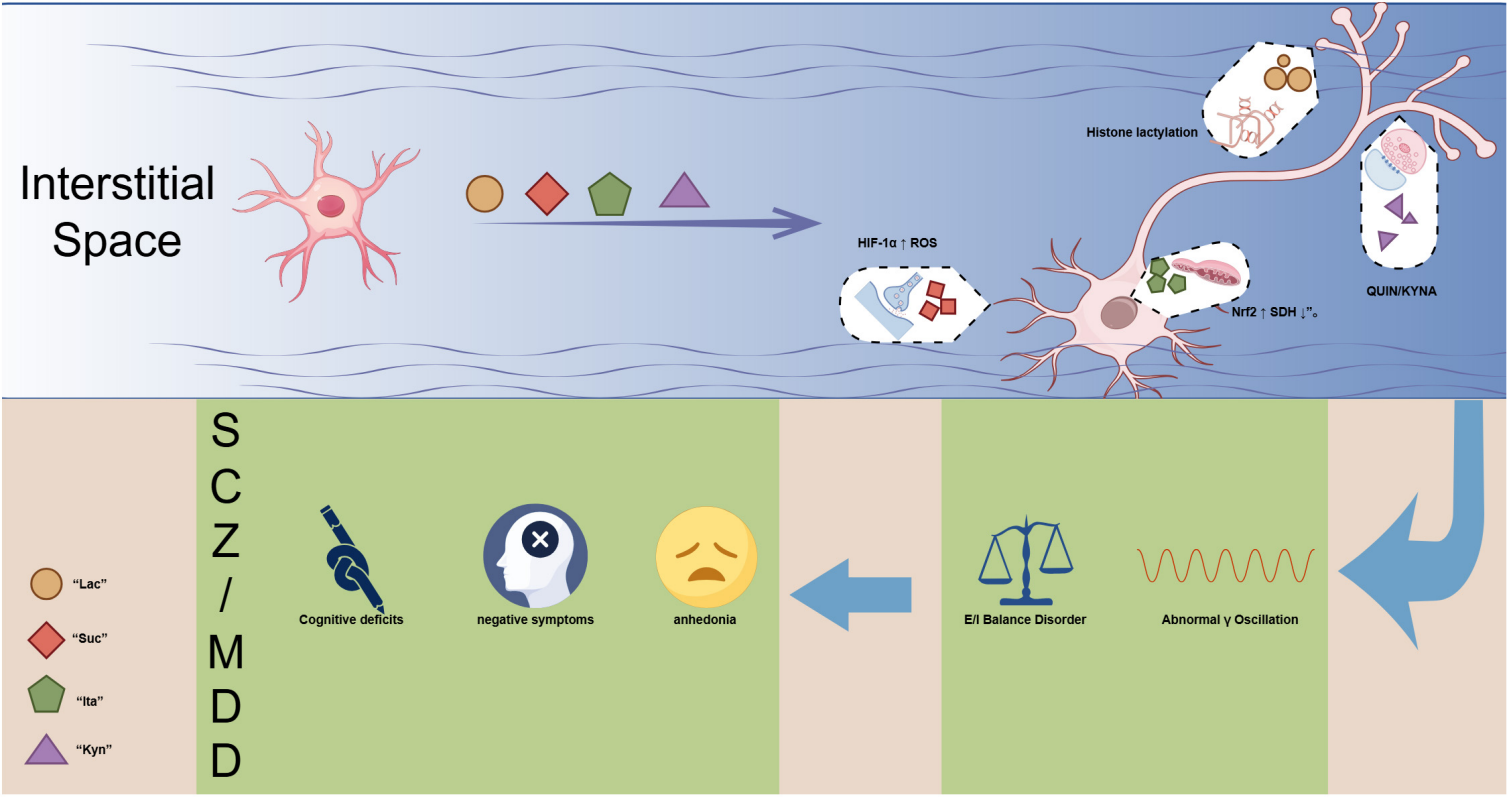
The neuro-immune-metabolic axis (NIMA): from microglial metabolites to circuit dysfunction and psychiatric disorders.

We synthesize current knowledge on the generation, signaling mechanisms, circuit-level effects, and behavioral impacts of microglia-derived metabolites, as summarized in Figure 1, which provides a schematic overview of the NIMA linking microglial metabolites to neural circuits, behaviors, and psychiatric disorders. We further discuss their therapeutic potential in psychiatric diseases.

## 2 The microglial signaling metabolite repertoire

### 2.1 Lactate

#### 2.1.1 Production, release, and epigenetic regulation

Upon neuroinflammatory activation, microglia undergo metabolic reprogramming characterized by a shift toward aerobic glycolysis (the Warburg effect), leading to substantial production and release of lactate (Pan et al., 2022). This lactate is shuttled into the interstitial space primarily via monocarboxylate transporters (MCTs) (Yang et al., 2025). Beyond serving as an energetic substrate, lactate functions as a signaling molecule capable of inducing histone lactylation—an emerging epigenetic modification that regulates the expression of immunometabolic and neuronal genes (Jiang et al., 2021; Zhang et al., 2019). In microglia, lactate-driven histone lactylation modulates transcriptional programs that influence inflammatory phenotype switching (Wang et al., 2019; Zhang et al., 2019). Thus, the cascade “inflammatory activation → glycolytic upregulation → lactate release → MCT-mediated transport → histone lactylation” constitutes a key mechanistic pathway linking microglial metabolism to epigenetic regulation in the brain.

#### 2.1.2 Impact on neuronal function

Lactate fine-tunes neuronal activity through dual, often bidirectional, mechanisms: energy provision and receptor- or epigenetically-mediated signaling. As an energy substrate, lactate supports high-frequency network oscillations and sustains synaptic transmission during heightened neuronal activity (Tang et al., 2014). In parallel, lactate acts via specific receptors such as the hydroxycarboxylic acid receptor 1 (HCAR1) to modulate neuronal excitability (Lauritzen et al., 2014; Tang et al., 2014). Additionally, through histone lactylation, lactate regulates genes involved in synaptic plasticity and calcium homeostasis (Liu and Zhou, 2024; Wang et al., 2019). This dual role enables lactate to exert concentration-dependent effects on neural synchronization: physiological levels support gamma oscillations (γ-oscillations), whereas pathological accumulation can disrupt network activity by impairing mitochondrial function and interneuron performance (Zhang et al., 2023). Consequently, lactate serves as a critical metabolic signal that integrates energy metabolism with the dynamic regulation of neuronal excitability and synaptic efficacy.

### 2.2 Succinate

#### 2.2.1 Generation, accumulation, and core signaling mechanisms

Succinate, a key intermediate in the tricarboxylic acid (TCA) cycle, accumulates under conditions of inflammatory activation or mitochondrial dysfunction due to metabolic remodeling, primarily characterized by suppressed succinate dehydrogenase (SDH) activity (Tannahill et al., 2013). This accumulation triggers two core signaling pathways. Intracellularly, succinate competitively inhibits α-ketoglutarate-dependent prolyl hydroxylases (PHDs), leading to stabilization of hypoxia-inducible factor-1α (HIF-1α) and subsequent transcription of pro-inflammatory and glycolytic genes, such as IL-1β (Tannahill et al., 2013). Extracellularly, upon release into the interstitial space, succinate can act in a paracrine manner by activating the SUCNR1 (GPR91) receptor on neighboring microglia, neurons, and astrocytes (Jia and Wang, 2025; Peruzzotti-Jametti et al., 2018). SUCNR1 activation initiates Gαi/Gαq-mediated signaling, resulting in ERK1/2 phosphorylation, NF-κB activation, and calcium mobilization, thereby amplifying neuroinflammatory signaling (Macias-Ceja et al., 2019; Peruzzotti-Jametti et al., 2018; Rothhammer et al., 2018; Rubic et al., 2008).

#### 2.2.2 Self-amplifying loops in neuroinflammation

Succinate potentiates neuroinflammatory responses through interconnected positive feedback loops that create a self-sustaining cycle (Maes et al., 2025). Intracellularly, HIF-1α stabilization not only promotes IL-1β expression but also enhances glycolytic flux, which can further contribute to succinate accumulation. Simultaneously, impaired SDH activity or reverse electron transport (RET) at mitochondrial complex II drives a burst of mitochondrial reactive oxygen species (mtROS) (Chahardehi et al., 2025; Mills et al., 2016; Weinberg and Chandel, 2025). This ROS surge activates the NLRP3 inflammasome, leading to caspase-1 activation and further maturation/secretion of IL-1β (Iwata et al., 2016; Maes et al., 2025). Extracellularly, SUCNR1 activation on immune cells stimulates additional release of inflammatory mediators like TNF-α (Li et al., 2024), which can in turn promote further succinate production and release from target cells. This vicious cycle—where succinate drives ROS and cytokine production, which then exacerbates succinate-associated signaling—chronically activates microglia and amplifies inflammatory cascades (Zhao et al., 2025). Ultimately, through sustained oxidative stress, pro-inflammatory cytokine toxicity, and disruption of metabolic homeostasis, this succinate-centered loop contributes significantly to synaptic dysfunction and neuronal damage, propagating neuropathology (Maes et al., 2025).

### 2.3 Itaconate

#### 2.3.1 Biosynthesis and core signaling mechanisms

Itaconate is an immunometabolite catalytically produced by ACOD1 (also known as IRG1) (Mills et al., 2018). It exerts potent anti-inflammatory and antioxidant effects through a dual core mechanism (Gao H. et al., 2025; Liu et al., 2025): (i) alkylation of KEAP1, which promotes Nrf2 stabilization and nuclear translocation to activate antioxidant transcriptional programs (Liu et al., 2025; Mills et al., 2018; Silva-Islas and Maldonado, 2018); and (ii) competitive inhibition of SDH, thereby reducing succinate accumulation, attenuating reverse electron transport (RET)-mediated mitochondrial reactive oxygen species (mtROS) generation, and dampening pro-inflammatory signaling (Gao H. et al., 2025; Lampropoulou et al., 2016; Mills et al., 2016). This positions itaconate as a key endogenous immunometabolic regulator (Moroni et al., 2012).

#### 2.3.2 Neuroprotective potential

Through these dual pathways—activating the Nrf2-mediated antioxidant response and inhibiting SDH-driven pro-inflammatory signaling—itaconate downregulates key pro-inflammatory cytokines and promotes a metabolic shift in microglia, driving their transition from a pro-inflammatory toward a repair phenotype (Bambouskova et al., 2018; Chen Y. J. et al., 2023; Mills et al., 2018; Runtsch et al., 2022). This reprogramming helps restore redox homeostasis, limits NLRP3 inflammasome activation, and protects neuronal structure and synaptic function (Simpson et al., 2007), underscoring its therapeutic potential in neuroinflammatory contexts (Zheng et al., 2023).

### 2.4 Kynurenine

#### 2.4.1 Production, release, and core signaling mechanisms

The kynurenine pathway (KP) serves as a major route of tryptophan metabolism, primarily regulated by the enzymes indoleamine 2,3-dioxygenase (IDO) and tryptophan 2,3-dioxygenase (TDO) (Stone, 2001). Pro-inflammatory signals potently induce IDO, shifting metabolism toward the production of neuroactive metabolites that are released into the interstitial space (Brum et al., 2023; O’Connor et al., 2009; Schwarcz et al., 2012). The signaling function of this pathway hinges on two key metabolites with opposing actions on the N-methyl-D-aspartate (NMDA) receptor: kynurenic acid (KYNA), an endogenous NMDA receptor antagonist (Moroni et al., 2012), and quinolinic acid (QUIN), an NMDA receptor agonist (Schwarcz et al., 1983; Stone, 2001). The precise balance between KYNA and QUIN within the interstitial compartment acts as a critical metabolic switch, fine-tuning glutamatergic transmission and neuronal excitability (Schwarcz et al., 2012; Tan et al., 2012).

#### 2.4.2 Impact on neuronal homeostasis and pathological imbalance

Physiologically, the equilibrium between KYNA and QUIN contributes to maintaining neuronal homeostasis, preventing both excessive excitation and insufficient glutamatergic tone (Stone, 2001). However, pathological disruption of this balance has significant consequences. A skew toward excess QUIN drives neuronal excitotoxicity and synaptic damage via sustained NMDA receptor overactivation, leading to calcium overload and oxidative stress (Behan et al., 1999; Cathomas et al., 2021). Conversely, elevated KYNA can induce glutamatergic hypofunction through excessive NMDA receptor blockade, thereby impairing synaptic plasticity and cognitive processes (Stachowski and Schwarcz, 2012; Stone, 2001). Thus, the KP constitutes a pivotal metabolic bridge linking immune activation to the precise regulation of central glutamatergic signaling and neuronal integrity (Table 1).

**TABLE 1:** Key signaling metabolites derived from microglia.

Metabolite	Core signaling mechanisms	Primary neural effects	Pathophysiological role
Lactate	•HCAR1 activation •Histone lactylation	•Energy substrate and modulator of excitability •Supports synaptic plasticity and gamma oscillations	High levels disrupt interneuron function and network synchrony (e.g., in Schizophrenia).
Succinate	•Intracellular: HIF-1α stabilization •Extracellular: SUCNR1 activation	•Amplifies pro-inflammatory responses (IL-1β) •Drives mtROS and NLRP3 activation	Sustains vicious cycles of neuroinflammation, leading to synaptic damage (e.g., in MDD).
Itaconate	•KEAP1 alkylation → Nrf2 activation •SDH inhibition	•Potent anti-inflammatory and antioxidant •Attenuates succinate-driven inflammation	Deficient signaling exacerbates inflammation; its analogs hold therapeutic potential.
Kynurenine pathway	•Balance between QUIN (NMDA receptor agonist) and KYNA (NMDA receptor antagonist)	•Fine-tunes glutamatergic transmission and excitability	KYNA-dominant shift: NMDA receptor hypofunction (e.g., in Schizophrenia). QUIN-dominant shift: excitotoxicity (e.g., in MDD).

## 3 From molecules to circuits

### 3.1 Coordinated regulation of synaptic plasticity and network oscillations

The metabolites discussed above do not act in isolation but constitute a dynamic regulatory network that operates in concert at both synaptic and network levels to shape neural circuit function. This integrated action can be conceptually organized into three functional modules: an energy and immediate signaling module (Yirmiya et al., 2015), an inflammation-excitotoxicity balancing module, and a protective and homeostatic restoration module. Together, these modules critically converge on two key circuit-level readouts frequently disrupted in psychiatric disorders: the excitation/inhibition (E/I) balance and the synchronization of neural networks, particularly γ-oscillations (30–40 Hz) which are essential for cognition and working memory.

#### 3.1.1 Energy metabolism and immediate signaling module (lactate, adenosine)

This module provides the rapid bioenergetic and signaling foundation required to sustain high-frequency synaptic transmission and network synchrony. Lactate, shuttled from glycolytically active microglia and astrocytes via MCTs, serves a dual role as an energetic substrate for neurons and a signaling molecule (Liu and Zhou, 2024; Tang et al., 2014; Yang et al., 2025). It supports the metabolic demands of active neurons, thereby facilitating synaptic potentiation and sustaining high-frequency network activity. Furthermore, lactate potentiates NMDA receptor function, promoting synaptic plasticity and associated gene expression, partly through epigenetic mechanisms like histone lactylation (Liu and Zhou, 2024; Zhang et al., 2019). Its effects on γ-oscillations are concentration-dependent: physiological levels help maintain neural synchrony, while pathological accumulation can disrupt oscillations by impairing mitochondrial function, particularly in parvalbumin-positive (PV+) interneurons crucial for generating rhythmic activity (Yang et al., 2014; Zhang et al., 2019, 2023). Complementing lactate, adenosine—derived from activity-dependent hydrolysis of ATP—acts as an inhibitory balancer. It fine-tunes synaptic activity primarily through presynaptic A1 receptor-mediated suppression of glutamate release and modulation of NMDA receptor function (Huang et al., 2011; Porkka-Heiskanen et al., 1997). This purinergic signaling is vital for preventing network hyperexcitability, regulating sleep-wake cycles, and ensuring synaptic homeostasis. Together, lactate and adenosine form a metabolic-signaling duo that underpins the immediate energy supply and dynamic inhibition necessary for normal synaptic plasticity and oscillatory dynamics.

#### 3.1.2 Inflammation-excitotoxicity balancing module (succinate, kynurenine pathway)

This module governs synaptic stability and survival by modulating the delicate equilibrium between neuroinflammation and excitatory tone. Succinate, a TCA cycle intermediate that accumulates during microglial activation or mitochondrial dysfunction, acts as a potent excitotoxicity and inflammation amplifier (Maes et al., 2025; Tannahill et al., 2013). Intracellularly, it stabilizes HIF-1α by inhibiting prolyl hydroxylases (PHDs), driving the expression of pro-inflammatory cytokines like IL-1β (Tannahill et al., 2013). Concurrently, it induces a burst of mitochondrial reactive oxygen species (mtROS) via reverse electron transport at SDH, further activating inflammatory cascades such as the NLRP3 inflammasome (Mills et al., 2016; Weinberg and Chandel, 2025). Extracellularly, succinate can engage the SUCNR1 receptor on neighboring cells, propagating pro-inflammatory signaling (Li et al., 2024; Peruzzotti-Jametti et al., 2018). The KP metabolites provide a complementary, bidirectional switch for glutamatergic transmission. The balance between its two key neuroactive products—QUIN (an NMDA receptor agonist) and KYNA (an NMDA receptor antagonist)—precisely tunes neuronal excitability and synaptic NMDA receptor function (Moroni et al., 2012; Stone, 2001; Tan et al., 2012). A pathological skew toward QUIN promotes excitotoxicity and calcium-mediated synaptic damage, while excess KYNA leads to NMDA receptor hypofunction, impairing synaptic plasticity (Schwarcz et al., 1983; Stachowski and Schwarcz, 2012; Stone, 2001). Thus, succinate and the KP converge on mechanisms involving oxidative stress, cytokine release, and NMDA receptor modulation to collectively determine synaptic fate—either supporting stability or driving degeneration.

#### 3.1.3 Protection and homeostatic restoration module (itaconate, neuroactive steroids)

Acting as a crucial counter-regulatory axis, this module provides negative feedback to limit inflammatory and oxidative damage, thereby promoting repair and synaptic resilience. Itaconate, an immunometabolite synthesized by activated microglia via the enzyme ACOD1/Irg1, exerts potent anti-inflammatory and antioxidant effects (Liu et al., 2025; Mills et al., 2018). Its mechanisms are 2-fold: first, it alkylates KEAP1 to activate the Nrf2 pathway, leading to the upregulation of antioxidant genes; second, it competitively inhibits SDH, thereby reducing succinate accumulation and the associated mtROS production and NLRP3 inflammasome activation (Gao H. et al., 2025; Lampropoulou et al., 2016; Mills et al., 2016). Through these actions, itaconate downregulates pro-inflammatory cytokines (e.g., IL-1β, TNF-α) and protects synaptic integrity. Neuroactive steroids, such as allopregnanolone, contribute to homeostasis by enhancing tonic inhibition via GABA_A receptor modulation, which can suppress microglial activation and constrain neuroinflammation, forming a stabilizing feedback loop (Marx et al., 2011; Pan et al., 2022). This module therefore restores redox balance, limits excitotoxic and inflammatory cascades, and creates a microenvironment conducive to synaptic recovery.

#### 3.1.4 Integrated effects on E/I balance and gamma oscillations

The coordinated actions of these metabolic modules ultimately converge to regulate two fundamental and interconnected circuit properties: the E/I balance and the synchronization of γ-oscillations. The energy/inhibition module (lactate, adenosine) directly fuels and finely tunes the activity of interneurons, particularly PV+ cells, which are pivotal for generating gamma rhythms and maintaining inhibitory tone (Lewis et al., 2012; Zhang et al., 2023). Dysregulation here—such as pathological lactate accumulation impairing interneuron metabolism or aberrant adenosine signaling—can directly disrupt γ-oscillations and shift the E/I balance toward hyper- or hypo-excitability. Simultaneously, the inflammation-excitotoxicity module (succinate, KP) can indirectly degrade these circuit properties. By promoting oxidative stress, neuroinflammation, and aberrant NMDA receptor signaling, this module compromises the health and function of the very interneurons that sustain oscillations, while also altering glutamatergic synaptic weights, thereby destabilizing the E/I equilibrium (Maes et al., 2025; Steullet et al., 2016; Tannahill et al., 2013). Finally, the protective module (itaconate, neurosteroids) serves to buffer these disruptive processes, restoring homeostasis and preserving the functionality of the circuits underlying synchronized activity. Therefore, the NIMA, through the integrated and often opposing influences of its constituent metabolites, exerts precise control over synaptic plasticity and network synchrony. Disruption of this coordinated regulation represents a core mechanism underlying the circuit dysfunction observed across psychiatric disorders.

### 3.2 The interstitial space: a determinant of spatiotemporal specificity in metabolite signaling

The signaling functions of microglia-derived metabolites are not executed in a void but within the dynamic and structured interstitial space of the brain (Tønnesen et al., 2018; Xie et al., 2013). This compartment, far from being a passive gap, acts as a critical signaling hub whose biophysical properties fundamentally govern the spatiotemporal profile of metabolic communication, thereby filtering and shaping the functional outcomes on neural circuits and behavior.

#### 3.2.1 Fundamental biophysical properties: diffusion, concentration, and signal lifetime

The interstitial space constitutes a fluid-filled network whose volume fraction and tortuosity determine the diffusion kinetics of signaling molecules (Syková and Nicholson, 2008; Xie et al., 2013). These physical parameters directly dictate a metabolite’s diffusion range, its achievable local concentration at target sites (e.g., synaptic clefts), and its functional lifetime before clearance (Hrabetova et al., 2018; Tønnesen et al., 2018). A smaller volume fraction or higher tortuosity restricts diffusion, leading to localized, high-concentration signaling niches. Conversely, a more open space facilitates broader dissemination but at potentially lower, sub-threshold concentrations. Thus, the interstitial architecture acts as a primary filter, selectively amplifying or attenuating microglial metabolic signals based on their molecular properties and the local extracellular geometry.

#### 3.2.2 Dynamicity and brain-state dependency: the “accumulation-clearance” cycle

A pivotal feature of the interstitial space is its dynamic fluctuation, tightly coupled to brain states, most notably the sleep-wake cycle (Xie et al., 2013). During active wakefulness, neuronal and glial activity leads to a relative contraction of the interstitial volume. This contraction favors the local accumulation of signaling metabolites released by microglia and other cells, such as lactate, which can then reach effective concentrations to modulate synaptic plasticity and network oscillations (Dienel, 2019; Wyss et al., 2011). Conversely, during slow-wave sleep, the interstitial space expands significantly (Xie et al., 2013). This expansion dramatically enhances the convective bulk flow of interstitial fluid, facilitating the glymphatic clearance of metabolic waste products and potentially neurotoxic or pro-inflammatory substances that have accumulated during wakefulness (Nedergaard and Goldman, 2020). This includes excitatory metabolites like QUIN and pro-inflammatory signals like succinate (Hablitz et al., 2019; Savitz, 2020).

This rhythmic “diurnal accumulation-nocturnal clearance” cycle is crucial for brain homeostasis. It allows for the beneficial signaling functions of metabolites like lactate during cognitive demand while preventing the pathological buildup of excitotoxic and inflammatory mediators during rest. The dysregulation of this cycle is directly implicated in psychiatric symptomatology. For instance, chronic sleep disruption or stress can pathologically constrict the interstitial space (Koundal et al., 2020; Kress et al., 2014). This impairment hinders the clearance of metabolites such as succinate. The resulting abnormal accumulation sustains pro-inflammatory signaling via receptors like SUCNR1, a mechanism strongly associated with depressive-like phenotypes in preclinical models (Gao T. et al., 2025). This breakdown links directly to clinical features: impaired clearance during sleep leads to the persistence of neuroinflammatory and excitatory tones, contributing to the daytime fatigue, cognitive slowing, and maladaptive synaptic plasticity observed in disorders like major depression (Carrard et al., 2018; Savitz et al., 2015).

#### 3.2.3 Regional heterogeneity: anatomical substrates for behavioral specificity

The properties of the interstitial space are not uniform across the brain, exhibiting significant regional heterogeneity in volume fraction, tortuosity, and the composition of the extracellular matrix (Hrabetova et al., 2018; Xie et al., 2013). This heterogeneity, combined with region-specific cellular metabolic demands and receptor expression patterns, creates distinct microenvironments that shape metabolite signaling. Consequently, identical amounts of a metabolite released by microglia can exert profoundly different effects in different brain circuits.

For example, the prefrontal cortex and the hippocampus display heightened sensitivity to lactate accumulation and KP dysregulation (Hagihara et al., 2018; Savitz et al., 2015). This regional vulnerability may stem from differences in interstitial architecture, local energy demands, or synaptic density, which affect metabolite diffusion and availability. Such specificity explains how metabolic disturbances can selectively impact circuits governing particular behaviors. Supporting this, spatial diffusion constraints significantly influence behavioral outcomes. In mice, the loss of microglial monocarboxylate transporter 4 (MCT4), a key lactate shuttling protein, leads to aberrant synaptic pruning and pronounced anxiety-like behaviors (Erblich et al., 2011; Sierra et al., 2014). This phenotype is likely due to disrupted lactate dynamics within the spatially constrained interstitial environment of prefrontal-limbic circuits, highlighting how the physical properties of the interstitial space gate the impact of metabolic signaling on specific behavioral domains like anxiety.

In summary, the interstitial space is a dynamic and heterogeneous regulator that confers spatiotemporal specificity to microglia-derived metabolite signals. Its physical properties control signal spread, its state-dependent volume changes enforce a vital clearance cycle, and its regional variability underlies the circuit- and behavior-specific effects of metabolic dysregulation. Disruptions at any level of this regulatory framework can drive the circuit dysfunction central to psychiatric disorders, making the interstitial space a critical conceptual and potential therapeutic node in the NIMA.

### 3.3 From dysregulated metabolic signaling to behavioral phenotypes

In summary, microglia-derived metabolites orchestrate higher-order cognitive and affective behaviors by fine-tuning neural circuit operations through the synergistic and spatiotemporally specific mechanisms described above. This regulation operates via a integrated “metabolite-interstitial space-circuit” signaling axis. Dysregulation of this axis manifests as three core behavioral pathological phenotypes: (Bohlen et al., 2017) cognitive impairments, such as deficits in working memory and executive function, linked to disrupted synaptic plasticity and network synchronization (e.g., aberrant γ-oscillations) (Lewis et al., 2012; Uhlhaas and Singer, 2010; Uhlhaas et al., 2008; Yang et al., 2025) affective dysregulation, including depression and anhedonia, associated with altered metabolite levels (e.g., lactate, KP metabolites) impacting prefrontal-limbic circuit integrity (Duman et al., 2016; Savitz et al., 2015; Savitz, 2020; Nayak et al., 2014) social dysfunction, arising from impaired microglial modulation of neural circuits governing social behavior (Zhan et al., 2014; Zhou et al., 2020). The following section will examine how disease-specific disruptions of particular metabolites selectively impair these related circuits, thereby giving rise to characteristic clinical symptom profiles in major psychiatric disorders.

## 4 Microglia-derived metabolites in major psychiatric disorders

### 4.1 Schizophrenia

#### 4.1.1 Characteristic alterations in metabolite profiles

Patients with SZ exhibit distinct metabolic disturbances in key microglia-derived signaling molecules, particularly involving lactate and the KP. Multi-omics studies have demonstrated that lactate levels are significantly elevated in the cerebrospinal fluid and brain parenchyma of individuals with SZ (Prabakaran et al., 2004). This elevation exhibits a robust correlation with the severity of negative symptoms and treatment resistance (Dogan et al., 2018). Concurrently, dysregulation of the KP plays a pivotal role, with a characteristic shift toward the neuroprotective branch. In SZ, the pathway exhibits a predominant bias toward KYNA accumulation (Pocivavsek et al., 2014; Stachowski and Schwarcz, 2012). Elevated levels of KYNA in the brain interstitial space are posited to underpin the glutamatergic hypofunction hypothesis of the disorder (Comai et al., 2025). These alterations are not mere epiphenomena but are integral to the disease pathophysiology, directly contributing to the emergence of cognitive and affective symptoms by disrupting neural circuit function.

#### 4.1.2 Mechanisms of circuit-level dysfunction

These metabolite imbalances drive neural circuit dysfunction through converging mechanisms that disrupt E/I balance and synaptic integrity. First, lactate accumulation, beyond its role as an energy substrate, may serve as a metabolic underpinning for aberrant γ-oscillations (Lewis et al., 2012; Liu and Zhou, 2024). PV+ interneurons, essential for generating synchronous gamma rhythms, are exquisitely sensitive to bioenergetic supply. Pathological lactate levels, potentially mediated through mechanisms such as impaired mitochondrial function or histone lactylation altering gene expression in these interneurons, can disrupt their fast-spiking activity (Marín, 2016; Prabakaran et al., 2004; Zhang et al., 2019). This impairs gamma oscillation generation, leading to desynchronization of prefrontal networks critical for working memory and contributing to the E/I imbalance observed in SZ (Foss-Feig et al., 2017; Steullet et al., 2016).

Second, the elevated KYNA levels directly impact synaptic plasticity and signal integration. By acting as an endogenous NMDA receptor antagonist, excessive KYNA exacerbates cortical glutamatergic hypofunction (Moroni et al., 2012; Stachowski and Schwarcz, 2012). This attenuates NMDA receptor-mediated currents, impairing mechanisms of synaptic plasticity such as long-term potentiation (LTP) that are fundamental for learning and cognitive function. The resultant dampening of glutamate signaling within prefrontal-limbic circuits disrupts information processing, contributing to the cognitive deficits and impaired executive function that are core features of SZ.

#### 4.1.3 Link to clinical symptomatology

The circuit dysfunctions orchestrated by these metabolic disturbances provide a mechanistic scaffold for the diverse clinical symptoms of SZ. Cognitive symptoms, particularly working memory impairments, are strongly linked to the disruption of γ-oscillations and E/I imbalance caused by lactate-mediated interneuron dysfunction and KYNA-induced NMDA receptor hypofunction (Lewis et al., 2012; Uhlhaas and Singer, 2010; Uhlhaas et al., 2009). Negative symptoms (e.g., avolition, blunted affect) may arise from a generalized prefrontal cortical hypofunction, driven by the combined impact of impaired energy metabolism (lactate dysregulation) and reduced glutamatergic tone (KYNA excess), leading to decreased neural activity and drive (Dogan et al., 2018; McIntyre et al., 2020). Positive symptoms (e.g., hallucinations, delusions) may involve more distributed circuit disturbances. Dysfunctional gamma synchrony and E/I imbalance could disrupt the fidelity of signal transmission in cortical-thalamic and temporal-prefrontal circuits, potentially leading to aberrant assignment of salience and impaired reality monitoring, which are theorized to underlie psychotic phenomena (Uhlhaas and Singer, 2010; Uhlhaas et al., 2008). Thus, microglial metabolic reprogramming and the resultant interstitial metabolite profile act as a critical node, linking cellular pathology to the spectrum of circuit dysfunctions that manifest as the complex clinical picture of SZ.

### 4.2 Major depressive disorder

#### 4.2.1 Kynurenine pathway and glutamatergic toxicity

Dysregulation of the KP plays a central role in the pathophysiology of MDD. Under pro-inflammatory conditions, cytokines induce the enzyme IDO, shifting tryptophan metabolism toward the neurotoxic branch of the KP and promoting the production of QUIN over KYNA (Hughes et al., 2024; Stone, 2001). In MDD, the pathway is characteristically skewed toward QUIN accumulation (Hughes et al., 2024; Owe-Young et al., 2008; Savitz et al., 2015). Acting as an agonist at the NMDA receptor, elevated interstitial QUIN induces excitotoxicity, particularly within prefrontal-limbic circuits such as the hippocampus and amygdala (Roeske et al., 2021; Schwarcz et al., 1983). This excessive NMDA receptor activation leads to calcium overload, oxidative stress, and synaptic damage (Cathomas et al., 2021). The resultant impairment in synaptic plasticity within these emotion- and cognition-processing networks is strongly implicated in the core symptomatology of MDD, including deficits in emotional regulation and cognitive domains such as memory and executive function (Aarsland et al., 2017; Stone, 2001). Thus, the inflammation-driven shift toward QUIN generation establishes a direct metabolic link between peripheral immune activation and central glutamatergic dysfunction, underpinning key affective and cognitive symptoms of depression.

#### 4.2.2 Succinate/itaconate and inflammatory loops

Neuroinflammation is a hallmark of MDD, wherein microglial-derived metabolites succinate and itaconate play opposing yet interconnected roles. Maes et al. (2025) propose succinate as a central node within the “inflammation-metabolism-mood disorder” axis implicated in depression. Under stress or inflammatory conditions, microglia undergo metabolic remodeling leading to succinate accumulation due to suppressed SDH activity (Tannahill et al., 2013). This accumulation triggers dual pro-inflammatory signaling: intracellularly, it stabilizes HIF-1α by inhibiting prolyl hydroxylases (PHDs), enhancing transcription of pro-inflammatory cytokines like IL-1β (Gao T. et al., 2025; Tannahill et al., 2013); extracellularly, it activates the SUCNR1 receptor on neighboring cells, further amplifying inflammatory cascades (Jia and Wang, 2025; Peruzzotti-Jametti et al., 2018). This process initiates a vicious cycle: succinate accumulation promotes mitochondrial ROS (mtROS) generation, which activates the NLRP3 inflammasome and upregulates IL-1β. This leads to sustained microglial activation, synaptic damage, dendritic spine loss, and a worsening of emotional and cognitive phenotypes (Mills et al., 2016; Schroder et al., 2010; Tannahill et al., 2013).

Conversely, itaconate serves as a crucial endogenous brake on this inflammatory loop. Produced by the enzyme ACOD1 (IRG1) in activated immune cells, itaconate exerts potent anti-inflammatory and antioxidant effects (Liu et al., 2025; Mills et al., 2018). It inhibits SDH, thereby reducing succinate accumulation and mtROS production, and alkylates KEAP1 to activate the Nrf2 antioxidant pathway (Liu et al., 2025; Mills et al., 2018). Through these mechanisms, itaconate downregulates pro-inflammatory cytokines (e.g., IL-1β, IL-6, TNF-α) and limits NLRP3 inflammasome activation (Bambouskova et al., 2018; Mills et al., 2018; Runtsch et al., 2022). In preclinical models of depression, itaconate ameliorates depressive-like behaviors and mitigates microglial activation and neuronal damage (Gao H. et al., 2025; Zheng et al., 2023). Therefore, the balance between the pro-inflammatory succinate axis and the protective itaconate axis is critical; a deficit in itaconate signaling or an overactive succinate pathway may heighten susceptibility to depression, whereas enhancing the itaconate-Nrf2 axis represents a promising therapeutic strategy (Haroon et al., 2017).

#### 4.2.3 Mood, motivation, and cognitive deficits

The metabolic disturbances described above converge to drive the core behavioral and cognitive deficits of MDD through distinct yet interconnected circuit mechanisms. First, KP dysregulation and QUIN-mediated excitotoxicity within the prefrontal cortex (PFC) and hippocampus impair synaptic plasticity and network synchrony, directly contributing to emotional dysregulation and cognitive dysfunction (Aarsland et al., 2017; Savitz, 2020). Second, succinate-driven neuroinflammation and oxidative stress further damage neuronal integrity and synaptic connections in limbic regions, exacerbating mood disturbances and motivation loss (Maes et al., 2025; Tannahill et al., 2013). Third, lactate metabolism abnormalities also contribute to depressive pathophysiology. In models of depression, aberrations in lactate metabolism coupled with microglial Kv1.3 channel activation are associated with exacerbated neuroinflammation and behavioral deterioration (Magistretti and Allaman, 2018; Pan et al., 2022). Lactate is essential for sustaining wakefulness and cognitive alertness by supporting high-frequency network oscillations and synaptic transmission in regions like the PFC (Carrard et al., 2018; Wyss et al., 2011). Diurnal dysregulation of lactate homeostasis may underlie the daytime fatigue and cognitive slowing observed in MDD. Compromised lactate metabolism in the PFC undermines the on-demand energy supply for cognitive effort, impairing executive function and motivation (Wyss et al., 2011). Finally, purinergic signaling adds another layer. Adenosine, accumulating via activity-dependent ATP hydrolysis, is a fundamental sleep regulator (Porkka-Heiskanen et al., 1997). Persistently heightened adenosinergic signaling in MDD may contribute to psychomotor retardation, profound fatigue, and the disruption of sleep-wake cycles (Huang et al., 2011). Collectively, these metabolite-driven disruptions—impairing PFC energy metabolism and function (motivation/executive function), damaging limbic synaptic plasticity (emotion), and dysregulating adenosine signaling (fatigue/arousal)—create a self-reinforcing cycle that sustains the multifaceted symptomatology of MDD.

### 4.3 Bipolar disorder, anxiety, and related disorders

Altered microglial metabolite signaling extends to bipolar disorder (BD) and anxiety disorders, underscoring the broad relevance of the NIMA. In BD, lactate dynamics are implicated in the neuroenergetic fluctuations underlying mood episodes. Shifts between manic and depressive states may involve altered astrocyte-microglia lactate shuttling, affecting prefrontal and limbic circuit energetics required for emotional regulation and cognitive stability (Carrard et al., 2018; McIntyre et al., 2020). Concurrent dysregulation of the KP, particularly elevations in neurotoxic QUIN during depressive phases, may exacerbate glutamatergic toxicity and neuroinflammation, contributing to mood instability and cognitive deficits (Savitz, 2020; Skaper et al., 2014).

In anxiety disorders, metabolite dysregulation converges on circuits governing fear and threat appraisal, such as the amygdala-prefrontal axis. KP imbalance, favoring either excessive QUIN (promoting excitotoxicity) or KYNA (inducing NMDA hypofunction), can disrupt amygdala excitability and prefrontal top-down control (Savitz, 2020; Ye et al., 2024). Furthermore, neurosteroids (e.g., allopregnanolone) and adenosine play significant roles. Allopregnanolone modulates GABA_A receptor function, and its deficit is linked to impaired stress resilience and anxiety-like behaviors (Pan et al., 2022). Adenosine signaling, particularly via A2B receptors, regulates sleep-wake cycles and neuronal excitability; its dysregulation may underlie the hyperarousal and sleep disturbances characteristic of anxiety disorders (Huang et al., 2011; Porkka-Heiskanen et al., 1997). In addiction, similar metabolites may influence rewards circuitry, with neurosteroids and adenosine modulating reinforcement and withdrawal behaviors (Pinna et al., 2005).

These observations suggest that despite distinct clinical presentations, disorders such as BD and anxiety may share a common pathophysiology wherein microglial metabolic reprogramming disrupts specific neural circuits—be it prefrontal-limbic energetics in BD or amygdala-prefrontal synchronization in anxiety. This highlights the generalizability of the microglia-metabolite-circuit framework across psychiatric conditions, pointing to convergent mechanisms that could be targeted for transdiagnostic therapeutic strategies (Lee et al., 2023; Table 2).

**TABLE 2:** Metabolic dysregulation across psychiatric disorders.

Disorder	Key metabolite alterations	Circuit and clinical correlates
Schizophrenia	•↑Lactate •↑KYNA (KP shift)	Circuit: PV+ interneuron dysfunction → impaired gamma oscillations; NMDA receptor hypofunction → impaired synaptic plasticity. Symptoms: cognitive deficits (working memory); negative symptoms.
Major depressive disorder	•↑QUIN (KP shift) •↑Succinate / ↓ Itaconate	Circuit: excitotoxicity in prefrontal-limbic circuits; neuroinflammation and oxidative stress. Symptoms: affective dysregulation; cognitive impairment; fatigue.
Bipolar Disorder	•Altered lactate dynamics •KP dysregulation (e.g., ↑ QUIN in depression)	Circuit: fluctuating energetics in prefrontal-limbic circuits; inflammation and excitotoxicity. Symptoms: mood instability; cognitive alterations.
Anxiety disorders	•KP imbalance •Altered neurosteroid/adenosine signaling	Circuit: disrupted amygdala excitability and prefrontal top-down control. Symptoms: hyperarousal; fear; sleep disturbances.

## 5 Therapeutic target prospects and future directions

### 5.1 Targeting the metabolite-interstitial space axis

Beyond targeting specific metabolic pathways or receptors, a promising therapeutic frontier lies in modulating the metabolite-interstitial space axis. This approach recognizes that the interstitial compartment is not a passive conduit but an active determinant of metabolite signaling, governing diffusion, concentration, and functional impact (Jessen et al., 2015; Tønnesen et al., 2018; Xie et al., 2013).

Targeting the signaling molecules themselves: This strategy focuses on directly modulating the key immunometabolic players within this axis. It includes developing agents to regulate critical enzymes—such as SDH, IDO/TDO, or ACOD1 (IRG1)—to control the flux of metabolites like succinate, kynurenine, and itaconate (Breda et al., 2016; Gao H. et al., 2025; Stone, 2001; Wang et al., 2022). Alternatively, pharmacologically targeting metabolite-sensing receptors—for example, using SUCNR1 antagonists to block pro-inflammatory succinate signaling or Nrf2 activators to boost the protective itaconate-KEAP1-Nrf2 axis—can selectively tune downstream inflammatory and oxidative stress pathways (Chen H. et al., 2023; Mills et al., 2018; Sun et al., 2022).

Modulating Interstitial Space Dynamics: Therapeutic intervention can also aim at optimizing the biophysical properties of the interstitial space to restore healthy metabolite dynamics (Jessen et al., 2015; Syková and Nicholson, 2008). Given the critical role of sleep in glymphatic clearance (Xie et al., 2013), improving sleep quality represents a non-pharmacological strategy to enhance the nocturnal washout of accumulated excitatory/pro-inflammatory metabolites (e.g., succinate, QUIN). Pharmacologically, targeting aquaporin-4 (AQP4) polarization on astrocytic endfeet may potentiate this clearance function (Iliff et al., 2012; Nedergaard and Goldman, 2020). Furthermore, developing agents that directly modulate the local microenvironment—such as those influencing extracellular matrix composition or regional blood flow—could help normalize interstitial volume and tortuosity, thereby correcting pathological metabolite accumulation and restoring physiological signaling gradients (Hrabetova et al., 2018; Koundal et al., 2020).

### 5.2 Chronotherapy and personalized metabolic interventions

Future therapeutic strategies should emphasize chrono-metabolic interventions—timing treatments to specific phases of the sleep-wake cycle to maximize efficacy. For example, promoting interstitial clearance of excitatory/inflammatory metabolites (e.g., succinate, QUIN) during sleep, while enhancing metabolic support (e.g., lactate shuttling) or antagonizing adenosine signaling during wakefulness, could restore neuro-immune-metabolic homeostasis more precisely. This approach, tailored to individual circadian and metabolic profiles, represents a promising frontier for personalized psychiatry (Koronowski and Sassone-Corsi, 2021; Walker et al., 2020; Wirz-Justice and Benedetti, 2020).

### 5.3 Technical challenges and future directions

Future research confronts pivotal technical challenges: the development of in vivo, real-time, high-resolution tools for monitoring metabolite and interstitial space dynamics, alongside brain region- and cell type-specific delivery systems (Marvin et al., 2018; Tønnesen et al., 2018).

## 6 Summary and conclusion

In conclusion, reconceptualizing microglia-derived metabolites as dynamic signaling molecules within the neuro-immune-metabolic axis establishes a transformative paradigm that fundamentally links cellular metabolism, neuroimmune crosstalk, neural circuit computation, and behavioral phenotypes. This integrated framework moves beyond traditional compartmentalized views, offering a unified mechanism through which metabolic reprogramming in microglia shapes synaptic plasticity, network synchronization, and ultimately higher-order cognition and emotion. Looking forward, elucidating the spatiotemporal specificity of this axis opens novel avenues for identifying circuit-specific metabolic biomarkers and developing precision interventions—ranging from metabolite-targeted pharmacology to chronotherapeutic modulation of interstitial clearance. Thus, targeting the microglial metabolite-interstitial space-neural circuit axis holds significant promise for advancing biomarker discovery and personalized therapeutic strategies in psychiatry.

## References

[B1] Aarsland D. Creese B. Politis M. Chaudhuri K. R. Ffytche D. H. Weintraub D. (2017). Cognitive decline in Parkinson disease. *Nat. Rev. Neurol.* 13 217–231. 10.1038/nrneurol.2017.27 28257128 PMC5643027

[B2] Bambouskova M. Gorvel L. Lampropoulou V. Sergushichev A. Loginicheva E. Johnson K. (2018). Electrophilic properties of itaconate and derivatives regulate the IκBζ-ATF3 inflammatory axis. *Nature* 556 501–504. 10.1038/s41586-018-0052-z 29670287 PMC6037913

[B3] Behan W. M. McDonald M. Darlington L. G. Stone T. W. (1999). Oxidative stress as a mechanism for quinolinic acid-induced hippocampal damage: Protection by melatonin and deprenyl. *Br. J. Pharmacol.* 128 1754–1760. 10.1038/sj.bjp.0702940 10588931 PMC1571800

[B4] Bernier L. P. York E. M. MacVicar B. A. (2020b). Immunometabolism in the brain: How metabolism shapes microglial function. *Trends Neurosci.* 43 854–869. 10.1016/j.tins.2020.08.008 32958333

[B5] Bernier L. P. York E. M. Kamyabi A. Choi H. B. Weilinger N. L. MacVicar B. A. (2020a). Microglial metabolic flexibility supports immune surveillance of the brain parenchyma. *Nat. Commun.* 11:1559. 10.1038/s41467-020-15267-z 32214088 PMC7096448

[B6] Bohlen C. J. Bennett F. C. Tucker A. F. Collins H. Y. Mulinyawe S. B. Barres B. A. (2017). Diverse requirements for microglial survival, specification, and function revealed by defined-medium cultures. *Neuron* 94 759–773.e8. 10.1016/j.neuron.2017.04.043. 28521131 PMC5523817

[B7] Breda C. Sathyasaikumar K. V. Sograte Idrissi S. Notarangelo F. M. Estranero J. G. Moore G. G. (2016). Tryptophan-2,3-dioxygenase (TDO) inhibition ameliorates neurodegeneration by modulation of kynurenine pathway metabolites. *Proc. Natl. Acad. Sci. U. S. A.* 113 5435–5440. 10.1073/pnas.1604453113 27114543 PMC4868470

[B8] Brum M. Nieberler M. Kehrwald C. Knopf K. Brunkhorst-Kanaan N. Etyemez S. (2023). Phase-and disorder-specific differences in peripheral metabolites of the kynurenine pathway in major depression, bipolar affective disorder and schizophrenia. *World J. Biol. Psychiatry* 24 564–577. 10.1080/15622975.2023.2169348 36648064

[B9] Carrard A. Elsayed M. Margineanu M. Boury-Jamot B. Fragnière L. Meylan E. M. (2018). Peripheral administration of lactate produces antidepressant-like effects. *Mol. Psychiatry* 23 392–399. 10.1038/mp.2016.179 27752076 PMC5794893

[B10] Cathomas F. Guetter K. Seifritz E. Klaus F. Kaiser S. (2021). Quinolinic acid is associated with cognitive deficits in schizophrenia but not major depressive disorder. *Sci. Rep.* 11:9992. 10.1038/s41598-021-89335-9 33976271 PMC8113521

[B11] Chahardehi A. M. Arefnezhad R. Rafei S. Arzhangzadeh A. Nasiri R. Rezaei-Tazangi F. (2025). The effect of malonate as a succinate dehydrogenase inhibitor on myocardial ischemia/reperfusion injury. *Cell Biol. Int.* 49 1545–1563. 10.1002/cbin.70079 40937615

[B12] Chausse B. Kakimoto P. A. Kann O. (2021). Microglia and lipids: How metabolism controls brain innate immunity. *Semin. Cell Dev. Biol.* 112 137–144. 10.1016/j.semcdb.2020.08.001 32807643

[B13] Chen H. Fu S. Li X. Shi M. Qian J. Zhao S. (2023). Microglial glutaminase 1 mediates chronic restraint stress-induced depression-like behaviors and synaptic damages. *Signal Transduct Target Ther.* 8:452. 10.1038/s41392-023-01699-8 38097558 PMC10721840

[B14] Chen Y. J. Li G. N. Li X. J. Wei L. X. Fu M. J. Cheng Z. L. (2023). Targeting IRG1 reverses the immunosuppressive function of tumor-associated macrophages and enhances cancer immunotherapy. *Sci. Adv.* 9:eadg0654. 10.1126/sciadv.adg0654 37115931 PMC10146892

[B15] Comai S. Manchia M. Bosia M. Miola A. Poletti S. Benedetti F. (2025). Moving toward precision and personalized treatment strategies in psychiatry. *Int. J. Neuropsychopharmacol.* 28:yaf025. 10.1093/ijnp/pyaf025 40255203 PMC12084835

[B16] Dienel G. A. (2019). Brain glucose metabolism: Integration of energetics with function. *Physiol. Rev.* 99 949–1045. 10.1152/physrev.00062.2017 30565508

[B17] Dogan A. E. Yuksel C. Du F. Chouinard V. A. Öngür D. (2018). Brain lactate and pH in schizophrenia and bipolar disorder: A systematic review of findings from magnetic resonance studies. *Neuropsychopharmacology* 43 1681–1690. 10.1038/s41386-018-0041-9 29581538 PMC6006165

[B18] Duman R. S. Aghajanian G. K. Sanacora G. Krystal J. H. (2016). Synaptic plasticity and depression: New insights from stress and rapid-acting antidepressants. *Nat. Med.* 22 238–249. 10.1038/nm.4050 26937618 PMC5405628

[B19] Erblich B. Zhu L. Etgen A. M. Dobrenis K. Pollard J. W. (2011). Absence of colony stimulation factor-1 receptor results in loss of microglia, disrupted brain development and olfactory deficits. *PLoS One* 6:e26317. 10.1371/journal.pone.0026317 22046273 PMC3203114

[B20] Erny D. Dokalis N. Mezö C. Castoldi A. Mossad O. Staszewski O. (2021). Microbiota-derived acetate enables the metabolic fitness of the brain innate immune system during health and disease. *Cell Metab.* 33 2260–2276.e7. 10.1016/j.cmet.2021.10.010. 34731656

[B21] Foss-Feig J. H. Adkinson B. D. Ji J. L. Yang G. Srihari V. H. McPartland J. C. (2017). Searching for cross-diagnostic convergence: Neural mechanisms governing excitation and inhibition balance in schizophrenia and Autism spectrum disorders. *Biol. Psychiatry* 81 848–861. 10.1016/j.biopsych.2017.03.005 28434615 PMC5436134

[B22] Gao H. Ding M. Liu Y. Wang Y. Zhao S. Chen J. (2025). Reprogramming immunity with itaconate: Metabolic mechanisms and therapeutic perspectives. *Inflamm. Res.* 74:128. 10.1007/s00011-025-02087-4 40956430

[B23] Gao T. Wang Z. Tabarak S. Yin Q. Lu L. (2025). The glymphatic system in psychiatric disorders: A new perspective. *Sci. Bull.* 70 3939–3942. 10.1016/j.scib.2025.05.015 40379518

[B24] Gao X. Yu M. Huang T. Ge Y. Gao J. (2025). Succinate contributes to Staphylococcus aureus skin infection by reactive oxygen species-hypoxic inducible factor 1α-glycolysis axis. *Microb. Pathog.* 204:107529. 10.1016/j.micpath.2025.107529 40185171

[B25] Hablitz L. M. Vinitsky H. S. Sun Q. Stæger F. F. Sigurdsson B. Mortensen K. N. (2019). Increased glymphatic influx is correlated with high EEG delta power and low heart rate in mice under anesthesia. *Sci. Adv.* 5:eaav5447. 10.1126/sciadv.aav5447 30820460 PMC6392807

[B26] Hagihara H. Catts V. S. Katayama Y. Shoji H. Takagi T. Huang F. L. (2018). Decreased brain pH as a shared endophenotype of psychiatric disorders. *Neuropsychopharmacology* 43 459–468. 10.1038/npp.2017.167 28776581 PMC5770757

[B27] Haroon E. Miller A. H. Sanacora G. (2017). Inflammation, glutamate, and glia: A trio of trouble in mood disorders. *Neuropsychopharmacology* 42 193–215. 10.1038/npp.2016.199 27629368 PMC5143501

[B28] Holland R. McIntosh A. L. Finucane O. M. Mela V. Rubio-Araiz A. Timmons G. (2018). Inflammatory microglia are glycolytic and iron retentive and typify the microglia in APP/PS1 mice. *Brain Behav. Immun.* 68 183–196. 10.1016/j.bbi.2017.10.017 29061364

[B29] Hrabetova S. Cognet L. Rusakov D. A. Nägerl U. V. (2018). Unveiling the extracellular space of the brain: From super-resolved microstructure to in vivo function. *J. Neurosci.* 38 9355–9363. 10.1523/JNEUROSCI.1664-18.2018 30381427 PMC6706003

[B30] Huang Z. L. Urade Y. Hayaishi O. (2011). The role of adenosine in the regulation of sleep. *Curr. Top. Med. Chem.* 11 1047–1057. 10.2174/156802611795347654 21401496

[B31] Hughes H. Brady L. J. Schoonover K. E. (2024). GABAergic dysfunction in postmortem dorsolateral prefrontal cortex: Implications for cognitive deficits in schizophrenia and affective disorders. *Front. Cell Neurosci.* 18:1440834. 10.3389/fncel.2024.1440834 39381500 PMC11458443

[B32] Iliff J. J. Wang M. Liao Y. Plogg B. A. Peng W. Gundersen G. A. (2012). A paravascular pathway facilitates CSF flow through the brain parenchyma and the clearance of interstitial solutes, including amyloid β. *Sci. Transl. Med.* 4:147ra111. 10.1126/scitranslmed.3003748 22896675 PMC3551275

[B33] Iwata M. Ota K. T. Li X. Y. Sakaue F. Li N. Dutheil S. (2016). Psychological stress activates the inflammasome via release of adenosine triphosphate and stimulation of the purinergic type 2X7 receptor. *Biol. Psychiatry* 80 12–22. 10.1016/j.biopsych.2015.11.026 26831917

[B34] Jauhar S. Johnstone M. McKenna P. J. (2022). Schizophrenia. *Lancet* 399 473–486. 10.1016/S0140-6736(21)01730-X 35093231

[B35] Jessen N. A. Munk A. S. Lundgaard I. Nedergaard M. (2015). The glymphatic system: A Beginner’s guide. *Neurochem. Res.* 40 2583–2599. 10.1007/s11064-015-1581-6 25947369 PMC4636982

[B36] Jia Y. Wang L. (2025). From mechanisms to diseases: The Succinate-GPR91 axis in cardiometabolic diseases. *J. Cardiovasc. Transl. Res.* 18 1298–1311. 10.1007/s12265-025-10670-7 40705203

[B37] Jiang J. Huang D. Jiang Y. Hou J. Tian M. Li J. (2021). Lactate modulates cellular metabolism through histone lactylation-mediated gene expression in non-small cell Lung cancer. *Front. Oncol.* 11:647559. 10.3389/fonc.2021.647559 34150616 PMC8208031

[B38] Koronowski K. B. Sassone-Corsi P. (2021). Communicating clocks shape circadian homeostasis. *Science* 371:eabd0951. 10.1126/science.abd0951 33574181 PMC8123919

[B39] Koundal S. Elkin R. Nadeem S. Xue Y. Constantinou S. Sanggaard S. (2020). Optimal mass transport with lagrangian workflow reveals advective and diffusion driven solute transport in the glymphatic system. *Sci. Rep.* 10:1990. 10.1038/s41598-020-59045-9 32029859 PMC7004986

[B40] Kress B. T. Iliff J. J. Xia M. Wang M. Wei H. S. Zeppenfeld D. (2014). Impairment of paravascular clearance pathways in the aging brain. *Ann. Neurol.* 76 845–861. 10.1002/ana.24271 25204284 PMC4245362

[B41] Lampropoulou V. Sergushichev A. Bambouskova M. Nair S. Vincent E. E. Loginicheva E. (2016). Itaconate links inhibition of succinate dehydrogenase with macrophage metabolic remodeling and regulation of inflammation. *Cell Metab.* 24 158–166. 10.1016/j.cmet.2016.06.004 27374498 PMC5108454

[B42] Lauritzen K. H. Morland C. Puchades M. Holm-Hansen S. Hagelin E. M. Lauritzen F. (2014). Lactate receptor sites link neurotransmission, neurovascular coupling, and brain energy metabolism. *Cereb. Cortex* 24 2784–2795. 10.1093/cercor/bht136 23696276

[B43] Lee S. Devanney N. A. Golden L. R. Smith C. T. Schwartz J. L. Walsh A. E. (2023). APOE modulates microglial immunometabolism in response to age, amyloid pathology, and inflammatory challenge. *Cell Rep.* 42:112196. 10.1016/j.celrep.2023.112196 36871219 PMC10117631

[B44] Lewis D. A. Curley A. A. Glausier J. R. Volk D. W. (2012). Cortical parvalbumin interneurons and cognitive dysfunction in schizophrenia. *Trends Neurosci.* 35 57–67. 10.1016/j.tins.2011.10.004 22154068 PMC3253230

[B45] Li C. Liu H. Li J. He X. Zhu H. Fu W. (2024). Molecular basis of ligand recognition and activation of the human succinate receptor SUCR1. *Cell Res.* 34 594–596. 10.1038/s41422-024-00984-7 38834763 PMC11291503

[B46] Liu S. Zhou S. (2024). Lactate: A new target for brain disorders. *Neuroscience* 552 100–111. 10.1016/j.neuroscience.2024.06.023 38936457

[B47] Liu Y. Jiang X. Zhuang S. Zhu L. Zhu B. Rui K. (2025). Itaconate: A key regulator of immune responses and potential therapeutic target for autoimmune and inflammatory diseases. *Autoimmun. Rev.* 24:103885. 10.1016/j.autrev.2025.103885 40683614

[B48] Macias-Ceja D. C. Ortiz-Masiá D. Salvador P. Gisbert-Ferrándiz L. Hernández C. Hausmann M. (2019). Succinate receptor mediates intestinal inflammation and fibrosis. *Mucosal Immunol.* 12 178–187. 10.1038/s41385-018-0087-3 30279517

[B49] Maes M. Almulla A. F. You Z. Zhang Y. (2025). Neuroimmune, metabolic and oxidative stress pathways in major depressive disorder. *Nat. Rev. Neurol.* 21 473–489. 10.1038/s41582-025-01116-4 40659853

[B50] Magistretti P. J. Allaman I. (2018). Lactate in the brain: From metabolic end-product to signalling molecule. *Nat. Rev. Neurosci.* 19 235–249. 10.1038/nrn.2018.19 29515192

[B51] Marín O. (2016). Developmental timing and critical windows for the treatment of psychiatric disorders. *Nat. Med.* 22 1229–1238. 10.1038/nm.4225 27783067

[B52] Martins-Ferreira R. Calafell-Segura J. Leal B. Rodríguez-Ubreva J. Martínez-Saez E. Mereu E. (2025). The Human Microglia Atlas (HuMicA) unravels changes in disease-associated microglia subsets across neurodegenerative conditions. *Nat. Commun.* 16:739. 10.1038/s41467-025-56124-1 39820004 PMC11739505

[B53] Marvin J. S. Scholl B. Wilson D. E. Podgorski K. Kazemipour A. Müller J. A. (2018). Stability, affinity, and chromatic variants of the glutamate sensor iGluSnFR. *Nat. Methods* 15 936–939. 10.1038/s41592-018-0171-3 30377363 PMC6394230

[B54] Marx C. E. Bradford D. W. Hamer R. M. Naylor J. C. Allen T. B. Lieberman J. A. (2011). Pregnenolone as a novel therapeutic candidate in schizophrenia: Emerging preclinical and clinical evidence. *Neuroscience* 191 78–90. 10.1016/j.neuroscience.2011.06.076 21756978

[B55] McIntyre R. S. Berk M. Brietzke E. Goldstein B. I. López-Jaramillo C. Kessing L. V. (2020). Bipolar disorders. *Lancet* 396 1841–1856. 10.1016/S0140-6736(20)31544-0 33278937

[B56] Mills E. L. Kelly B. Logan A. Costa A. S. H. Varma M. Bryant C. E. (2016). Succinate dehydrogenase supports metabolic repurposing of mitochondria to drive inflammatory macrophages. *Cell* 167 457–470.e13. 10.1016/j.cell.2016.08.064. 27667687 PMC5863951

[B57] Mills E. L. Ryan D. G. Prag H. A. Dikovskaya D. Menon D. Zaslona Z. (2018). Itaconate is an anti-inflammatory metabolite that activates Nrf2 via alkylation of KEAP1. *Nature* 556 113–117. 10.1038/nature25986 29590092 PMC6047741

[B58] Moroni F. Cozzi A. Sili M. Mannaioni G. (2012). Kynurenic acid: A metabolite with multiple actions and multiple targets in brain and periphery. *J. Neural Transm*. 119 133–139. 10.1007/s00702-011-0763-x 22215208

[B59] Nayak D. Roth T. L. McGavern D. B. (2014). Microglia development and function. *Annu. Rev. Immunol.* 32 367–402. 10.1146/annurev-immunol-032713-120240 24471431 PMC5001846

[B60] Nedergaard M. Goldman S. A. (2020). Glymphatic failure as a final common pathway to dementia. *Science* 370 50–56. 10.1126/science.abb8739 33004510 PMC8186542

[B61] O’Connor J. C. Lawson M. A. André C. Briley E. M. Szegedi S. S. Lestage J. (2009). Induction of IDO by bacille Calmette-Guérin is responsible for development of murine depressive-like behavior. *J. Immunol.* 182 3202–3212. 10.4049/jimmunol.0802722 19234218 PMC2664258

[B62] Owe-Young R. Webster N. L. Mukhtar M. Pomerantz R. J. Smythe G. Walker D. (2008). Kynurenine pathway metabolism in human blood-brain-barrier cells: Implications for immune tolerance and neurotoxicity. *J. Neurochem.* 105 1346–1357. 10.1111/j.1471-4159.2008.05241.x 18221377

[B63] Pan R. Y. He L. Zhang J. Liu X. Liao Y. Gao J. (2022). Positive feedback regulation of microglial glucose metabolism by histone H4 lysine 12 lactylation in Alzheimer’s disease. *Cell Metab.* 34 634–648.e6. 10.1016/j.cmet.2022.02.013. 35303422

[B64] Peruzzotti-Jametti L. Bernstock J. D. Vicario N. Costa A. S. H. Kwok C. K. Leonardi T. (2018). Macrophage-Derived extracellular succinate licenses neural stem cells to suppress chronic neuroinflammation. *Cell Stem Cell.* 22 355–368.e13. 10.1016/j.stem.2018.01.020. 29478844 PMC5842147

[B65] Pinna G. Costa E. Guidotti A. (2005). Changes in brain testosterone and allopregnanolone biosynthesis elicit aggressive behavior. *Proc. Natl. Acad. Sci. U. S. A.* 102 2135–2140. 10.1073/pnas.0409643102 15677716 PMC548579

[B66] Pocivavsek A. Thomas M. A. Elmer G. I. Bruno J. P. Schwarcz R. (2014). Continuous kynurenine administration during the prenatal period, but not during adolescence, causes learning and memory deficits in adult rats. *Psychopharmacology* 231 2799–2809. 10.1007/s00213-014-3452-2 24590052 PMC4074218

[B67] Porkka-Heiskanen T. Strecker R. E. Thakkar M. Bjorkum A. A. Greene R. W. McCarley R. W. (1997). Adenosine: A mediator of the sleep-inducing effects of prolonged wakefulness. *Science* 276 1265–1268. 10.1126/science.276.5316.1265 9157887 PMC3599777

[B68] Prabakaran S. Swatton J. E. Ryan M. M. Huffaker S. J. Huang J. T. Griffin J. L. (2004). Mitochondrial dysfunction in schizophrenia: Evidence for compromised brain metabolism and oxidative stress. *Mol. Psychiatry* 9 684–697, 643. 10.1038/sj.mp.4001511. 15098003

[B69] Roeske M. J. McHugo M. Vandekar S. Blackford J. U. Woodward N. D. Heckers S. (2021). Incomplete hippocampal inversion in schizophrenia: Prevalence, severity, and impact on hippocampal structure. *Mol. Psychiatry* 26 5407–5416. 10.1038/s41380-020-01010-z 33437006 PMC8589684

[B70] Rothhammer V. Borucki D. M. Tjon E. C. Takenaka M. C. Chao C. C. Ardura-Fabregat A. (2018). Microglial control of astrocytes in response to microbial metabolites. *Nature* 557 724–728. 10.1038/s41586-018-0119-x 29769726 PMC6422159

[B71] Rubic T. Lametschwandtner G. Jost S. Hinteregger S. Kund J. Carballido-Perrig N. (2008). Triggering the succinate receptor GPR91 on dendritic cells enhances immunity. *Nat. Immunol.* 9 1261–1269. 10.1038/ni.1657 18820681

[B72] Runtsch M. C. Angiari S. Hooftman A. Wadhwa R. Zhang Y. Zheng Y. (2022). Itaconate and itaconate derivatives target JAK1 to suppress alternative activation of macrophages. *Cell Metab.* 34 487–501.e8. 10.1016/j.cmet.2022.02.002. 35235776

[B73] Savitz J. (2020). The kynurenine pathway: A finger in every pie. *Mol. Psychiatry* 25 131–147. 10.1038/s41380-019-0414-4 30980044 PMC6790159

[B74] Savitz J. Drevets W. C. Wurfel B. E. Ford B. N. Bellgowan P. S. Victor T. A. (2015). Reduction of kynurenic acid to quinolinic acid ratio in both the depressed and remitted phases of major depressive disorder. *Brain Behav. Immun.* 46 55–59. 10.1016/j.bbi.2015.02.007 25686798 PMC4414807

[B75] Schroder K. Zhou R. Tschopp J. (2010). The NLRP3 inflammasome: A sensor for metabolic danger? *Science* 327 296–300. 10.1126/science.1184003 20075245

[B76] Schwarcz R. Bruno J. P. Muchowski P. J. Wu H. Q. (2012). Kynurenines in the mammalian brain: When physiology meets pathology. *Nat. Rev. Neurosci.* 13 465–477. 10.1038/nrn3257 22678511 PMC3681811

[B77] Schwarcz R. Whetsell W. O. Mangano R. M. (1983). Quinolinic acid: An endogenous metabolite that produces axon-sparing lesions in rat brain. *Science* 219 316–318. 10.1126/science.6849138 6849138

[B78] Sierra A. Beccari S. Diaz-Aparicio I. Encinas J. M. Comeau S. Tremblay M. È (2014). Surveillance, phagocytosis, and inflammation: How never-resting microglia influence adult hippocampal neurogenesis. *Neural Plast.* 2014:610343. 10.1155/2014/610343 24772353 PMC3977558

[B79] Silva-Islas C. A. Maldonado P. D. (2018). Canonical and non-canonical mechanisms of Nrf2 activation. *Pharmacol. Res.* 134 92–99. 10.1016/j.phrs.2018.06.013 29913224

[B80] Simpson I. A. Carruthers A. Vannucci S. J. (2007). Supply and demand in cerebral energy metabolism: The role of nutrient transporters. *J. Cereb. Blood Flow Metab.* 27 1766–1791. 10.1038/sj.jcbfm.9600521 17579656 PMC2094104

[B81] Skaper S. D. Facci L. Giusti P. (2014). Neuroinflammation, microglia and mast cells in the pathophysiology of neurocognitive disorders: A review. *CNS Neurol. Disord. Drug Targets* 13 1654–1666. 10.2174/1871527313666141130224206 25470401

[B82] Stachowski E. K. Schwarcz R. (2012). Regulation of quinolinic acid neosynthesis in mouse, rat and human brain by iron and iron chelators in vitro. *J. Neural Transm.* 119 123–131. 10.1007/s00702-011-0694-6 21833493 PMC3670827

[B83] Steullet P. Cabungcal J. H. Monin A. Dwir D. O’Donnell P. Cuenod M. (2016). Redox dysregulation, neuroinflammation, and NMDA receptor hypofunction: A &quot;central hub&quot; in schizophrenia pathophysiology? *Schizophr. Res.* 176 41–51. 10.1016/j.schres.2014.06.021 25000913 PMC4282982

[B84] Stone T. W. (2001). Endogenous neurotoxins from tryptophan. *Toxicon* 39 61–73. 10.1016/s0041-0101(00)00156-2 10936623

[B85] Sun G. Zhang R. Liu C. Meng W. Pang Q. (2022). Itaconate attenuates neuroinflammation and exerts dopamine neuroprotection in parkinson’s disease through inhibiting NLRP3 inflammasome. *Brain Sci.* 12:1255. 10.3390/brainsci12091255 36138991 PMC9496935

[B86] Syková E. Nicholson C. (2008). Diffusion in brain extracellular space. *Physiol. Rev.* 88 1277–1340. 10.1152/physrev.00027.2007 18923183 PMC2785730

[B87] Tan L. Yu J. T. Tan L. (2012). The kynurenine pathway in neurodegenerative diseases: Mechanistic and therapeutic considerations. *J. Neurol. Sci.* 323 1–8. 10.1016/j.jns.2012.08.005 22939820

[B88] Tang F. Lane S. Korsak A. Paton J. F. Gourine A. V. Kasparov S. (2014). Lactate-mediated glia-neuronal signalling in the mammalian brain. *Nat. Commun.* 5:3284. 10.1038/ncomms4284 24518663 PMC3926012

[B89] Tannahill G. M. Curtis A. M. Adamik J. Palsson-McDermott E. M. McGettrick A. F. Goel G. (2013). Succinate is an inflammatory signal that induces IL-1β through HIF-1α. *Nature* 496 238–242. 10.1038/nature11986 23535595 PMC4031686

[B90] Tønnesen J. Inavalli V. V. G. K. Nägerl U. V. (2018). Super-Resolution Imaging of the Extracellular Space in Living Brain Tissue. *Cell* 172 1108–1121.e15. 10.1016/j.cell.2018.02.007. 29474910

[B91] Uhlhaas P. J. Singer W. (2010). Abnormal neural oscillations and synchrony in schizophrenia. *Nat. Rev. Neurosci.* 11 100–113. 10.1038/nrn2774 20087360

[B92] Uhlhaas P. J. Haenschel C. Nikoliæ D. Singer W. (2008). The role of oscillations and synchrony in cortical networks and their putative relevance for the pathophysiology of schizophrenia. *Schizophr. Bull.* 34 927–943. 10.1093/schbul/sbn062 18562344 PMC2632472

[B93] Uhlhaas P. J. Pipa G. Lima B. Melloni L. Neuenschwander S. Nikoliæ D. (2009). Neural synchrony in cortical networks: History, concept and current status. *Front. Integr. Neurosci.* 3:17. 10.3389/neuro.07.017.2009 19668703 PMC2723047

[B94] Walker W. H. Walton J. C. DeVries A. C. Nelson R. J. (2020). Circadian rhythm disruption and mental health. *Transl. Psychiatry* 10:28. 10.1038/s41398-020-0694-0 32066704 PMC7026420

[B95] Wang J. Cui Y. Yu Z. Wang W. Cheng X. Ji W. (2019). Brain endothelial cells maintain lactate homeostasis and control adult hippocampal neurogenesis. *Cell Stem Cell* 25 754–767.e9. 10.1016/j.stem.2019.09.009. 31761722

[B96] Wang Q. Lu M. Zhu X. Gu X. Zhang T. Xia C. (2022). The role of microglia immunometabolism in neurodegeneration: Focus on molecular determinants and metabolic intermediates of metabolic reprogramming. *Biomed. Pharmacother.* 153:113412. 10.1016/j.biopha.2022.113412 36076537

[B97] Weinberg S. E. Chandel N. S. (2025). Mitochondria reactive oxygen species signaling-dependent immune responses in macrophages and T cells. *Immunity* 58 1904–1921. 10.1016/j.immuni.2025.07.012 40763730 PMC12371701

[B98] Wirz-Justice A. Benedetti F. (2020). Perspectives in affective disorders: Clocks and sleep. *Eur. J. Neurosci.* 51 346–365. 10.1111/ejn.14362 30702783

[B99] Wyss M. T. Jolivet R. Buck A. Magistretti P. J. Weber B. (2011). In vivo evidence for lactate as a neuronal energy source. *J. Neurosci.* 31 7477–7485. 10.1523/JNEUROSCI.0415-11.2011 21593331 PMC6622597

[B100] Xie L. Kang H. Xu Q. Chen M. J. Liao Y. Thiyagarajan M. (2013). Sleep drives metabolite clearance from the adult brain. *Science* 342 373–377. 10.1126/science.1241224 24136970 PMC3880190

[B101] Yang H. Mo N. Tong L. Dong J. Fan Z. Jia M. (2025). Microglia lactylation in relation to central nervous system diseases. *Neural Regen. Res.* 20 29–40. 10.4103/NRR.NRR-D-23-00805 38767474 PMC11246148

[B102] Yang J. Ruchti E. Petit J. M. Jourdain P. Grenningloh G. Allaman I. (2014). Lactate promotes plasticity gene expression by potentiating NMDA signaling in neurons. *Proc. Natl. Acad. Sci. U. S. A.* 111 12228–12233. 10.1073/pnas.1322912111 25071212 PMC4143009

[B103] Ye F. Dong M. C. Xu C. X. Jiang N. Chang Q. Liu X. M. (2024). Effects of different chronic restraint stress periods on anxiety- and depression-like behaviors and tryptophan-kynurenine metabolism along the brain-gut axis in C57BL/6N mice. *Eur. J. Pharmacol.* 965:176301. 10.1016/j.ejphar.2023.176301 38145646

[B104] Yirmiya R. Rimmerman N. Reshef R. (2015). Depression as a microglial disease. *Trends Neurosci.* 38 637–658. 10.1016/j.tins.2015.08.001 26442697

[B105] Zhan Y. Paolicelli R. C. Sforazzini F. Weinhard L. Bolasco G. Pagani F. (2014). Deficient neuron-microglia signaling results in impaired functional brain connectivity and social behavior. *Nat. Neurosci.* 17 400–406. 10.1038/nn.3641 24487234

[B106] Zhang D. Tang Z. Huang H. Zhou G. Cui C. Weng Y. (2019). Metabolic regulation of gene expression by histone lactylation. *Nature* 574 575–580. 10.1038/s41586-019-1678-1 31645732 PMC6818755

[B107] Zhang Y. M. Qi Y. B. Gao Y. N. Chen W. G. Zhou T. Zang Y. (2023). Astrocyte metabolism and signaling pathways in the CNS. *Front. Neurosci.* 17:1217451. 10.3389/fnins.2023.1217451 37732313 PMC10507181

[B108] Zhao L. Li T. Dang M. Li Y. Lu J. Lu Z. (2025). Succinate Dehydrogenase Subunit A (SDHA) mediated microglia extracellular traps formation participating in cerebral ischemic reperfusion injury. *Adv. Sci.* 12:e11873. 10.1002/advs.202411873 40832885 PMC12463099

[B109] Zheng L. Wu S. Jin H. Wu J. Wang X. Cao Y. (2023). Molecular mechanisms and therapeutic potential of icariin in the treatment of Alzheimer’s disease. *Phytomedicine* 116:154890. 10.1016/j.phymed.2023.154890 37229892

[B110] Zhou H. Wang J. Zhang Y. Shao F. Wang W. (2020). The role of microglial CX3CR1 in schizophrenia-related behaviors induced by social isolation. *Front. Integr. Neurosci.* 14:551676. 10.3389/fnint.2020.551676 33013335 PMC7500158

